# The Retina in Patients With Triple A Syndrome: A Window Into Neurodegeneration?

**DOI:** 10.3389/fendo.2021.729056

**Published:** 2021-11-12

**Authors:** Fiorenza Ulgiati, Sophie Lhoir, Irina Balikova, Sylvie Tenoutasse, Emese Boros, Catheline Vilain, Claudine Heinrichs, Cécile Brachet

**Affiliations:** ^1^ Paediatric Endocrinology Unit – Hôpital Universitaire des Enfants Reine Fabiola, Université Libre de Bruxelles, Brussels, Belgium; ^2^ Pediatric Ophthalmology Unit, Hôpital Universitaire des Enfants Reine Fabiola, Université Libre de Bruxelles, Brussels, Belgium; ^3^ Department of Genetics, Hôpital Universitaire des Enfants Reine Fabiola, Université Libre de Bruxelles (ULB) Center of Human Genetics, Université Libre de Bruxelles, Brussels, Belgium

**Keywords:** AAAS, ALADIN, Triple A syndrome, OCT, retina, neurodegeneration, Redox, RNFL

## Abstract

**Objective:**

Experimental evidence suggests that the clinical manifestations of Triple A syndrome result from oxidative stress. Several conditions caused by oxidative stress display retinal involvement. Our objective was to assess the retina and optic nerve involvement in children with Triple A syndrome.

**Methods:**

Eleven patients with genetically proven Triple A syndrome followed-up in our centre were approached for study participation. The main outcome was the measurement of the thicknesses of the different retinal layers by Optical Coherence Tomography (OCT).

**Results:**

9 patients with triple A syndrome had OCT measurements. 7 patients were children and 2 were adults; 4 were females and 5 were males. The 7 paediatric patients had at least two OCT measured at a mean interval of 7.9 months after the first one. The average Retinal Nerve Fibre Layer thickness was 74 ± 10 µm in patients compared to the paediatric reference range of 100 ± 2 µm (p<0.05).

**Conclusions and Relevance:**

This is the first study to document retinal layer thicknesses in a series of patients with Triple A syndrome. Nearly all retinal thickness and peripapillary RNFL measurements were very significantly inferior to the reference range in Triple A patients, whatever their age. RNFL thinning was more marked at the temporal part of the optic nerve. OCT being non-invasive, it represents a promising tool to assess the severity of neurodegeneration in patients with Triple A syndrome.

## Introduction

Triple A syndrome or Allgrove syndrome is characterised by ACTH-resistant Adrenal insufficiency, Alacrima and Achalasia plus diverse neurological deficits. It was first described by Allgrove in 1978 (1). This autosomal recessive condition results from biallelic inactivation of the AAAS gene, which encodes a nuclear pore protein called ALADIN (alacrima–achalasia–adrenal insufficiency neurologic disorder) (2, 3). Pathogenic AAAS mutations lead either to absent ALADIN or to aberrant subcellular localisation of the ALADIN protein and nuclear pore complex malfunction. The nuclear pore complex regulates molecular trafficking between the nucleus and the cytoplasm (4).

In patients with triple A syndrome, alacrima is usually present since birth but the other features of the syndrome develop over time, pointing towards a degenerative process damaging the adrenal glands and the neuromuscular control of the lower esophageal sphincter along with other components of the nervous system. Increased oxidative stress has been shown to lead to increased apoptosis and to evolving tissue damage in patients with triple A syndrome. *In vitro* studies have documented oxidative stress in cultured adrenal cells, neuronal cells and fibroblasts harbouring *AAAS* mutations (5, 6). In patients’ fibroblasts, altered induction or downregulation of genes associated with oxidative stress and antioxidant defence have been shown (7). Furthermore, ALADIN interacts with the ferritin heavy chain protein (FTH1), which, in addition to its iron storage role, protects the nucleus from oxidative damage (8). Fibroblasts from triple A patients lack nuclear FTH1, suggesting that the nuclear translocation of FTH1 is defective. ALADIN has also been shown to interact directly with progesterone receptor membrane component 2 (PGRMC2), a microsomal protein known to regulate cytochrome P450 hydroxylases and oxidoreductases, which are critical for maintaining the cellular oxidative state and for generating precursors for hormones like cortisol (9). *In vivo*, a study in one patient revealed oxidative stress, evidenced by lipid peroxidation and LDL oxidation measurements (10).

Optical coherence tomography (OCT) is an imaging technique that allows quantitative imaging of the ten layers of the retina (11). Furthermore, it is particularly suitable for the paediatric population because of its non-invasiveness and short duration. The potential role of OCT measurements as biomarkers of disease progression in neurodegenerative disorders such as multiple sclerosis, Alzheimer’s disease, neuromyelitis optica and Parkinson’s disease, has been widely investigated (12–15).

Following the observation that one young patient followed for triple A syndrome presented poor colour vision, optic nerve atrophy was diagnosed. Some case reports in the literature had reported the findings of optic nerve paleness or colour vision loss in patients with triple A syndrome (16–19). We therefore decided to measure optic nerve thickness with OCT in all the patients followed-up for triple A syndrome by our team.

The aim of our study is to assess retinal involvement by OCT in patients with Triple A syndrome and to discuss the role of these measurements as markers of disease severity and progression.

## Patients and Methods

The study was approved by the ethical committee of Hôpital Universitaire des Enfants Reine Fabiola.

### Study Design

The study is a single-centre retrospective study. The patients or their caregiver consented to publication of medical information about them or their relative.

### Participants

The study included all patients with a genetically confirmed Triple A syndrome followed-up in HUDERF or previously followed-up in this Hospital between 1988 and mars 2021 and still in contact with the same medical team because of family relationship to an affected patient.

### Ophthalmological Examination

All patients underwent a thorough ophthalmic examination, in order to exclude ocular pathology that could potentially confound OCT analysis. This included measurement of best-corrected visual acuity with age-adapted optotype, colour vision assessment with Ishihara colour plates (colour vision was said to be abnormal if one or more Ishihara plate was incorrectly identified), pupil assessment, automated visual field screening, slit lamp bio microscopy, stereoscopic fundus examination with pupil dilation and digital retinal photography. The Heidelberg Spectralis (Heidelberg Engineering, Heidelberg, Germany) spectral domain (SD) OCT was used to obtain three high-resolution Macula-Fast scans and peripapillary retinal nerve fibre layer (RNFL) scans and retinal map for both eyes of each participant. The RNFL scan images the retinal nerve fibre layer in a 3.4 mm diameter circle centred on the optic nerve head. The Macula-Fast scan is used to assess the thickness and volume in the macular region. This scan images a 6 mm circle centred on the fovea. The area is divided into three circles, measuring 1, 3 and 6 mm in diameter: the two outer circles are divided into four quadrants (superior, nasal, inferior and temporal) and the central circle corresponds to the foveal region. The scans were obtained by one examiner in a single session. Care was taken to ensure that all scans were well centred. Both eyes were examined for each patient.

### Statistics

Continuous variables are presented as mean ± standard deviation (SD). Normative reference ranges as established by Perez-Garcia and colleagues in 2016 are used to compare to the measurements of the RNFL and macular thickness of our pædiatric study population (20). Normative reference ranges as established by Lu Cheng and colleagues are used to compare to the measurements of the ganglion cell layer and the inner plexiform layer of our pædiatric study population (21). Normative reference ranges as published in the Heidelberg Spectralis RNFL thickness database (including 330 eyes enrolled in Canada, the United States, and Germany and enrolling diverse study populations) is used to compare to the measurements of the RNFL and macular thickness of the two adult patients studied (Heidelberg Engineering GmbH 2016). Comparison of the mean ± SD of the varied measurements in our study population with the mean of the reference population was done using the one sample mean Z-test, the variance of the control sample being known. A p value below 0.05 was considered significant.

## Results

Eleven patients with Triple A syndrome currently or previously followed in our Paediatric Endocrinology and Ophthalmology Units were identified (**Table 1**).

**Table 1:** Characteristics of the study series of patients with Triple A syndrome.

n	Sex	Country of Origin	Genotype	Age at diagnosis (years)	Diagnostic circumstances
1	m	Morocco	C.1097 del et	3,4	Hypoglycaemia
			C.1331+1G>A		
2	m	Morocco	C.1097 del et	2.0	Family segregation
			C.l331+1G>A		
3	m	Morocco	C.658 T>C	3,3	Hypoglycaemia
			C.658 T>C		
4	m	Morocco	C.1331+1G>A	2, 2	Hypoglycaemia
			C.l331+1G>A		
5	f	Morocco	C.1331+1G>A	1,1	Family segregation
			C.l331+1G>A		
6	m	Morocco	C.1331+1G>A	3,0	Hypoglycaemia
			C.1331+1G>A		
7	f	Morocco	C.l331+1G>A	5,6	Vomiting
			C.1331+1G>A		
8	f	Morocco	C.1024 C>T	5,0	Family segregation
			C.1024 C>T		
9	f	Morocco	C.1024 C>T	9,5	Hypoglycaemia
			C.1024 C>T		
10	m	Morocco	C.1024 C>T	3,8	Hypoglycaemia
			C.1024 C>T		
11	m	Turkey	C.43C>A	6	Hypoglycaemia
			C.43C>A		

Patients 1-9 underwent OCT examinations. In grey, patients from the same family (sibling pairs for patients 1 and 2 and 4 and 5 and daughter and mother pair for patients 8 and 9). Note the pseudodominant presentation in the mother and daughter pair, because of multi-generational consanguinity.

The 11 patients come from 6 families, 5 of Moroccan origin and one of Turkish origin. Most of the patients (7/11) have been diagnosed after an acute adrenal crisis with severe hypoglycaemia, whereas one patient presented with achalasia, and the remaining were identified after family segregation study. All the patients were treated for ACTH-resistant adrenal insufficiency with hydrocortisone and 6/11 patients were also treated for mineralocorticoid deficiency with 9-αFludrocortisone with good treatment adherence. Alacrima was treated with artificial tears with moderate adherence. Six patients had had surgical treatment for achalasia. OCT measurement could not be performed in two patients because of loss of follow up in one case and severe neurological sequelae in the second case. Of the patients who participated, 7 patients were children and 2 were adults; 4 were females and 5 were males. Age (range) at first OCT analysis was 4 to 39 years. All 7 pædiatric patients had a second OCT measured at a mean interval of 7.9 months after the first one and 6/7 pædiatric patients had a third OCT measured at a mean interval of 9.3 months after the second one.

### Ophthalmological Examination

**Table 2** shows results from the ophthalmological evaluation in relation with the OCT findings. Eight patients out of 9 had temporal papillary pallor at fundus examination (not performed in one patient because of neurologic sequelae). Visual acuity was abnormal in all patients ranging from 0.5 to 0.85. Ishihara’s Test for Colour Deficiency could be reliably performed in 6 out of 9 patients. Colour vision was normal in one patient. It could not be performed in 4 patients because of loss of follow-up, severe neurological sequelae, or poor cooperation. Automated visual field screening was performed in 5 patients out of 9. Central scotoma was suspected in 4 patients. It was considered unreliable in 3 patients because of misunderstanding of the test. All patients had superficial punctuated keratitis at Slit lamp examination.

**Table 2:** Ophthalmological data and school type of the 9 patients with Triple A syndrome whose neurological status allowed an in-depth investigation.

Patient Number	Age at last OCT (y)	Temporal RNFL(µm)	Visual Acuity (right-left)	Visual Field	Colour Vision	School Type
1	9	27,00	0,6-0,7	central alterations	abnormal	Special needs
2	7.2	28,00	0,8-0,7	central alterations	–	Special needs
3	12.4	26,50	0,7-0,8	left central scotoma	abnormal	Special needs
4	13.7	25,50	0,8-0,7	central alterations	abnormal	Special needs
5	6.5	26,50	0,8-0,7	–	abnormal	Special needs
6	22.4	26,50	0,5-0,6	–	–	Special needs
7	6.2	24,50	0,8-0,8	–	–	Special needs
8	10.9	38,00	0,6-0,6	–	fair	Normal
9	38.6	39,00	0,5-0,6	–	abnormal	Special needs

OCT measurements in the patient series (both eyes) compared to the reference population (mean±SD).

### OCT Measurements

#### Peripapillary RNFL

**Figure 1:**
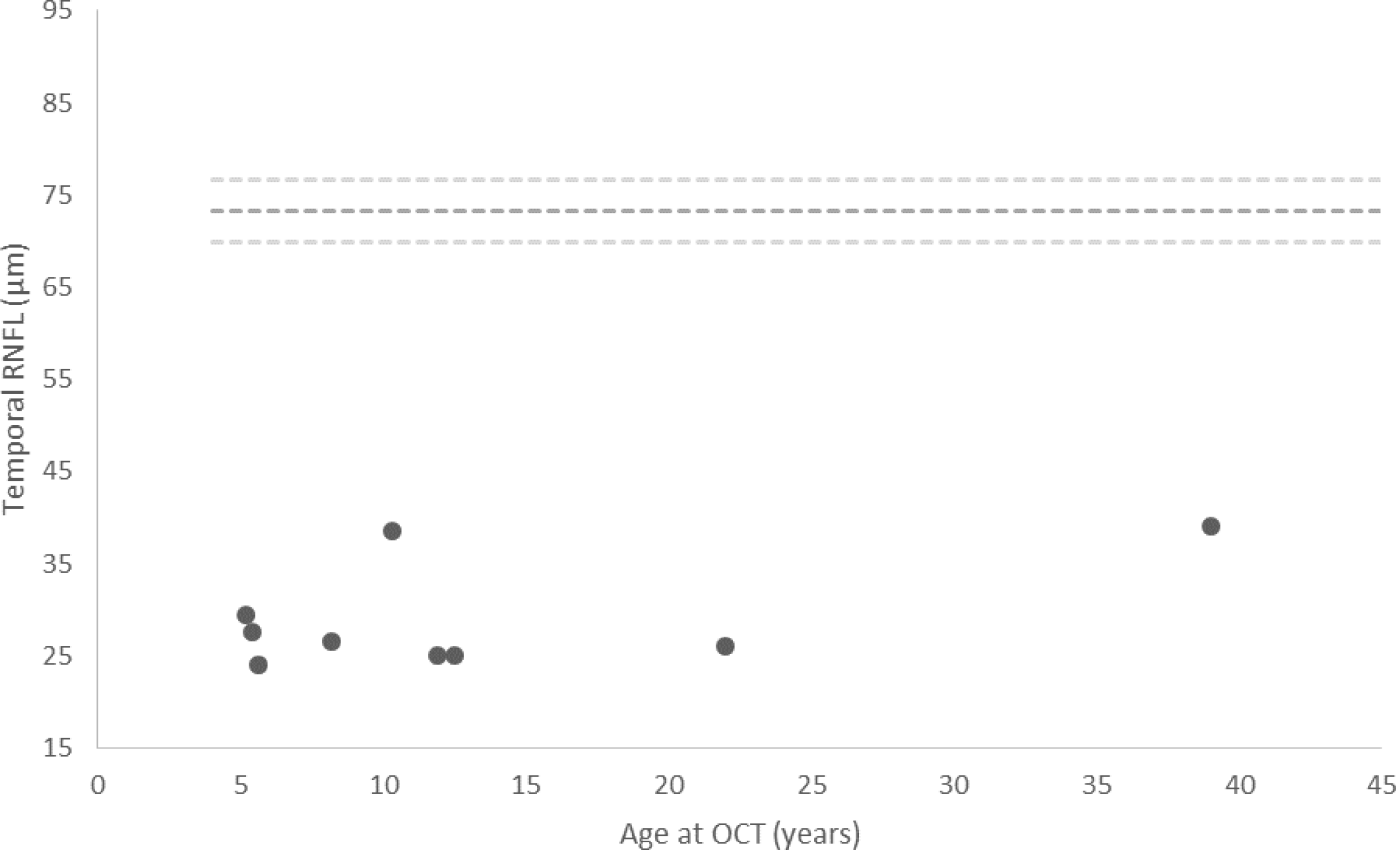
Temporal RNFL thickness measured at initial OCT in patients with Triple A syndrome. Each value is the mean between two eyes. The dashed lines represent the mean ± 2SD of the reference range (20). Of note, the outliers that display a temporal RNFL of nearly 40 µm are the mother and daughter pair.

Three out of 108 measurements were excluded from RNFL analysis at first OCT and 2 out of 108 measurements were excluded at second OCT, due to poor scan quality secondary to poor fixation. RNFL thickness was significantly lower in the patients compared to the mean of the reference paediatric population in 5 out of the 6 quadrants and markedly lower in the temporal quadrants (p<0.01) (**Table 3**). **Figure 1** shows the temporal RNFL according to age at first OCT in the patients.

**Table 3:** Retinal Nerve Fibre Layer thickness (RNFL); macular ganglion cell layer plus macular inner plexiform layer thickness (GCL+IPL).

	Pædiatric patients n = 7	Pædiatric reference	Adult patients n = 2	Adult reference
**Retinal Nerve Fibre Layer**				
Average RNFL (µm)	74 ± 10*	100 ± 2	74 ± 3	98 ± 9
Nasal superior RNFL (µm)	119 ± 14*	108 ± 3.4	112 ± 8	111 ± 15
Nasal RNFL (µm)	70 ± 19*	72 ± 1.9	70 ± 11	81 ± 12
Nasal inferior RNFL (µm)	88 ± 16*	112 ± 3.6	92 ± 29	113 ± 18
Temporal inferior RNFL (µm)	90 ± 16*	148 ± 2.9	69 ± 17	133 ± 15
Temporal RNFL (µm)	28 ± 5*	73 ± 1.7	30 ± 8	66 ± 12
Temporal superior RNFL (µm)	98 ± 23*	141 ± 2.8	114 ± 15	135 ± 19
**Macular thickness (µm)**				
Superior inner (µm)	312 ± 13*	345 ± 2.3	297 ± 6	346
Nasal inner (µm)	315 ± 11*	344 ± 2.3	305 ± 5	333
Inferior inner (µm)	306 ± 12*	341 ± 2.2	297 ± 12	340
Temporal inner (µm)	296 ± 10*	331 ± 2.2	290 ± 13	348
**GCL+IPL**				
Superior inner (µm)	51 ± 6*	94 ± 7		
Nasal inner (µm)	48 ± 5*	94 ± 7		
Inferior inner (µm)	49 ± 6*	94 ± 6		
Temporal inner (µm)	43 ± 4*	90 ± 6		

References: (20) for RNFL and macular thickness; (21) for GCL+IPL; (Heidelberg Engineering GmbH 2016) for adult reference (Heidelberg Engineering GmbH 2016)(Heidelberg Engineering GmbH 2016)(Heidelberg Engineering GmbH 2016) *p < 0.01 (one sample mean Z-test).

#### Macular Thickness Measurement

Macular thickness was significantly reduced in the patients compared to the mean of the reference paediatric population in the four quadrants of the macula (**Table 3**).

Macular ganglion cell layer (GCL) thickness and macular inner plexiform layer (IPL) thickness was significantly reduced in the patients compared to the mean of the reference paediatric population in the four quadrants of the macula (**Table 3**).

#### Repeated OCT Measurements

**Figure 2:**
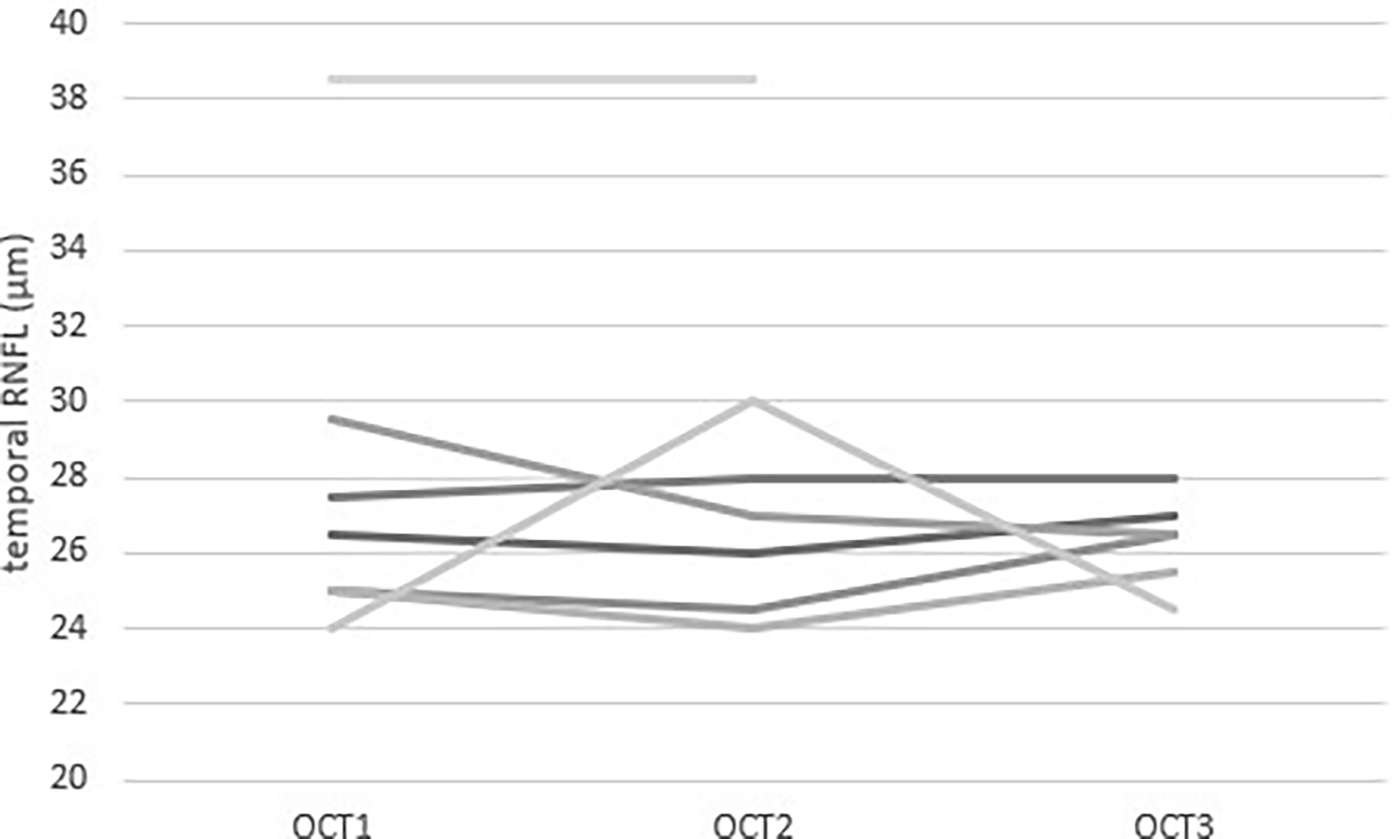
Repeated measurements of Temporal RNFL thickness measured by OCT in 7 paediatric patients with Triple A syndrome. Each value is the mean of the two eyes of one patient. Each line represents a patient. Three patients had three measurements during their follow-up. The mean time between OCT1 and OCT2 was 7.9 months and the mean time between OCT2 and OCT3 was 9.3 months.

OCT measurements were repeated in the paediatric patients after a mean interval of 7.9 months between the first and the second OCT and 9.3 months between the second and the third OCT (**Figure 2**).

## Discussion

This is the first study to document retinal layer thicknesses in patients with Triple A syndrome. Nearly all retinal thickness measurements are significantly lower in patients with Triple A syndrome than in the control cohorts, regardless of the age of the patients. The retinal thinning is more marked at the temporal region of the retina. The retinal thinning does not seem to be a rapidly progressive process.

Optic atrophy is rare in children. In patients with Triple A syndrome, it has been previously described in two teenage siblings (18) earlier reports mention optic disk paleness in a 21 year-old male (19), two 13 and 16 year-old boys (20) and two 3 and 6 year-old children (21).

Triple A syndrome is thought to result from an increased oxidative stress, as documented by *in vitro* and *in vivo* studies. Deficiency of ALADIN Impairs Redox Homeostasis *in vitro* in Adrenal and neuronal cells. (22) and markers of oxidative stress have been measured in a child with Triple A syndrome (10).

The sensitivity of adrenal glands to oxidative damage, is illustrated by three different rare conditions having in common adrenal insufficiency and oxidative stress: mutations in NNT, TXNRD2, or AAAS (22). Steroidogenesis is affected by oxidative stress through different mechanisms (23). The nervous system is also very sensitive to Redox disequilibrium. Mitochondrial dysfunction leading to oxidative stress and increased apoptosis is a feature of several inherited neurodegenerative disorders and OCT has been used to monitor disease progression in these disorders (24). Optic nerve involvement, particularly the preferential loss of the temporal RNFL, the nerve fibres which transmit central and colour vision, is a recognised feature of many mitochondrial disorders, including mitochondrial optic atrophy (25). This loss of temporal retinal ganglion cells and their axons in mitochondrial disease is attributed to the disadvantageous energy conditions of the small parvocellular axons (26). The small retinal ganglion cells in the macula may atrophy as the disease progresses and their axons, which emanate from the macula, run to the temporal aspect of the optic nerve (the papillo-macular bundle is either directly affected by the disease or becomes atrophic as a result of damage to the cell bodies in the macula) (25).

The preferential loss of temporal RNFL observed in our case series of patients with Triple A syndrome is similar to what is observed in mitochondrial optic neuropathies.

This finding, added to the documented oxidative damage observed in AAAS knock-down of adrenal and neuronal cell lines, are strong arguments for the implication of a redox disequilibrium in the retinal thinning we observed.

Our study’s has limitations: first, the small sample size (18 eyes included in OCT analysis, repeated twice in most patients, three times in some of them) due to the extreme rarity of the disease. To address this, it would be interesting to expand these measurements to a larger cohort of patients through an EndoERN collaboration. Another limitation regards the assessment of retinal thinning progression. The timeframe of our study is probably too short given that most neurological deficits progress rather slowly in patients with Triple A syndrome.

In conclusion, OCT being a rapid, objective, reproducible and non-invasive investigation method to document neural degeneration, it might have a clinical usefulness in children with Triple A syndrome to monitor neurodegeneration, as in other neurodegenerative diseases.

In addition, it could allow to assess the efficacy of potential therapeutic agents. In patients with triple A syndrome, the only publication about a disease-modifying agent regards N-acetylcysteine, a well-known antioxidant agent. *In vitro*, after knock-down of the AAAS gene, adrenal and neuronal cells displayed an increased viability after N-acetylcysteine treatment (5). *In vivo*, N-acetylcysteine (600 mg twice daily) has been administered to one paediatric patient in order to document its effect on oxidative stress measurements. The oxidative stress was assessed by measurements of a lipid peroxidation product, thiobarbituric acid reactive substances in plasma and measurement of reduced glutathione in whole blood. N-acetylcysteine was shown to lower lipid peroxidation products and increase reduced glutathione levels in the patient (5, 10). Our study documenting severe thinning of the retinal layers in children and adults with Triple A syndrome points towards OCT as a useful follow-up tool in these patients.

## Data Availability Statement

The original contributions presented in the study are included in the article/supplementary material. Further inquiries can be directed to the corresponding author.

## Ethics Statement

The studies involving human participants were reviewed and approved by Ethics Committee of Queen Fabiola Children’s University Hospital. Written informed consent from the participants’ legal guardian/next of kin was not required to participate in this study in accordance with the national legislation and the institutional requirements.

## Author Contributions

All authors listed have made a substantial, direct, and intellectual contribution to the work and approved it for publication.

## Conflict of Interest

The authors declare that the research was conducted in the absence of any commercial or financial relationships that could be construed as a potential conflict of interest.

## Publisher’s Note

All claims expressed in this article are solely those of the authors and do not necessarily represent those of their affiliated organizations, or those of the publisher, the editors and the reviewers. Any product that may be evaluated in this article, or claim that may be made by its manufacturer, is not guaranteed or endorsed by the publisher.
